# Quantifying the Impact of the Peptide Identification Framework on the Results of Fast Photochemical Oxidation of Protein Analysis

**DOI:** 10.1021/acs.jproteome.3c00390

**Published:** 2023-12-29

**Authors:** Marek Zakopcanik, Daniel Kavan, Petr Novak, Dmitry S. Loginov

**Affiliations:** † Institute of Microbiology, The Czech Academy of Sciences, 14220 Prague, Czech Republic; ‡ Department of Biochemistry, Faculty of Science, Charles University, 12820 Prague, Czech Republic

**Keywords:** FPOP, search engine, structural proteomics

## Abstract

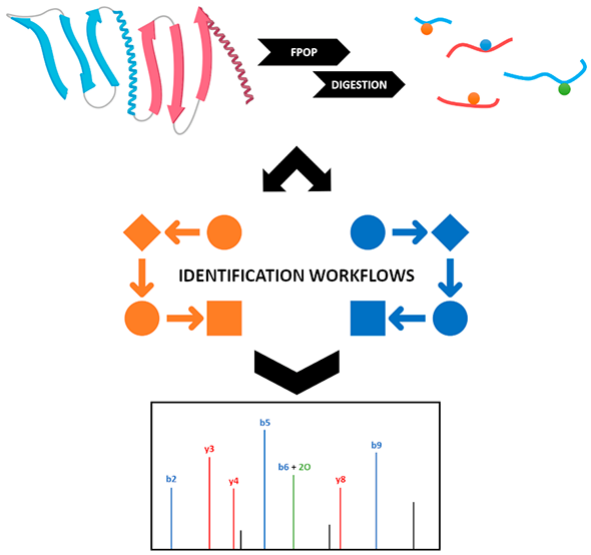

Fast Photochemical Oxidation of Proteins (FPOP) is a promising technique for studying protein structure and dynamics. The quality of insight provided by FPOP depends on the reliability of the determination of the modification site. This study investigates the performance of two search engines, Mascot and PEAKS, for the data processing of FPOP analyses. Comparison of Mascot and PEAKS of the hemoglobin–-haptoglobin Bruker timsTOF data set (PXD021621) revealed greater consistency in the Mascot identification of modified peptides, with around 26% of the IDs being mutual for all three replicates, compared to approximately 22% for PEAKS. The intersection between Mascot and PEAKS results revealed a limited number (31%) of shared modified peptides. Principal Component Analysis (PCA) using the peptide-spectrum match (PSM) score, site probability, and peptide intensity was applied to evaluate the results, and the analyses revealed distinct clusters of modified peptides. Mascot showed the ability to assess confident site determination, even with lower PSM scores. However, high PSM scores from PEAKS did not guarantee a reliable determination of the modification site. Fragmentation coverage of the modification position played a crucial role in Mascot assignments, while the AScore localizations from PEAKS often become ambiguous because the software employs MS/MS merging.

## Introduction

Information about the higher-order structure of proteins is vital for our understanding of their function. Mass spectrometry-based methods offer a wide range of approaches to study protein structure,^1^ interactions,^2^ and dynamics.^3^ One of these approaches is the covalent labeling of proteins. Covalent labeling might be implemented by different means. For example, the common technique hydrogen/deuterium exchange (HDX) relies on the exchange of amide hydrogens on the backbone of the protein.^4^ The efficiency of this method of labeling is based on solvent accessibility and the local hydrogen bonding network. HDX already has a well-established workflow and numerous data analysis software packages.^5−8^ Other methods of protein footprinting use reactive radical species such as fluoroalkyl radicals,^9,10^ carbonate radical,^11^ carbene diradical,^12^ and hydroxyl radical.^13^

Fast Photochemical Oxidation of Proteins (FPOP) employs labeling of amino acid side chains by hydroxyl radicals.^13^ The covalent modification of the residues is irreversible. Moreover, the ^•^OH properties are similar to those of the H_2_O molecules, offering a biologically relevant image of the protein state.^14^ The nonspecific nature of this reaction offers theoretically no limit in coverage, but in practice, the difference in reactivity between the amino acid side chains and the ^•^OH varies by 10^3^,^14,15^ resulting in the predominance of more reactive residue modifications (Cys, Met, Trp, Tyr, Phe, His) over less reactive (aliphatic and other polar) amino acids.^14^ Therefore, the extent of modification is not proportional to just the solvent accessibility but depends on the side chain reactivity as well. Furthermore, high confidence in the modification position is necessary for reliable and relevant results. While FPOP continues to develop, most recently via a top-down proteomic approach,^16,17^ the bottom-up approach is most commonly used.^18,19^ Currently, FPOP analysis is held back by tedious data processing, and the workflow has not yet been unified like in the case of HDX. A recent protocol by Liu et al.^20^ introduces guidelines to establish an FPOP platform but is limited in the number of modifications used and relies on Byos (Protein Metrics, CA, USA), which includes the FPOP workflow as part of the suite and cannot be obtained separately. One of the other proprietary programs used for FPOP data analysis is Sequest, but it is restricted in the number of possible modifications, thus requiring separate consecutive searches.^21^ The recently introduced algorithm update for cloud-based search engine Bolt promises an increased number of modifications while decreasing the processing time^22^ but so far lacks adaptation. There are two open source programs that might be used for FPOP data processing: MSS-clean^23^ and PepFoot.^24^ Their primary designation is footprinting experiments with a specific covalent probe, thus being able to run analysis with only one variable mass shift at a time. MSFragger,^25^ Comet,^26^ and MS-GF+^27^ are standard open source proteomic search engines that allow enough variable modifications to be suitable for FPOP, but they require employment of further processing modules to provide certainty assessment for the modification site.

The steps of FPOP data processing involve a database search, identification of the compounds from the MS/MS spectra, assignment of chromatographic peaks, and quantification of the signal intensity from the LC chromatogram. As previously published,^28^ the choice of search engine can significantly influence the identification of modified peptides in a data set. We decided to explore this aspect of data processing for FPOP analyses. Two representatives of different algorithmic approaches were chosen for this experiment: Mascot,^29^ as a purely database search-based software, and PEAKS,^30^ which employs *de novo* sequencing as a workflow improvement. Mascot searches the MS/MS data provided in the form of a peak list against a database of choice. Each peptide-spectrum match (PSM) is assigned a score based on the probability that the observed match is a random event. Confidence in the localization of the peptide modification is expressed as a percentage value derived from the difference in score between the two most probable sites.^31^ The number of variable modifications that can be defined for a search is limited to 9. PEAKS accepts raw analysis files from a variety of vendors (Agilent, Bruker, Thermo, and Waters) and runs its own data refinement. In pursuit of more reliable identification, PEAKS performs additive merging of spectra based on the precursor *m*/*z* and time window. Furthermore, it combines *de novo* and database search results together with additional features as an input for the internal Linear Discrimination Function to derive the *p*-value and ultimately score.^30^ Modification localization is reported as an AScore, reflecting the probability of the reported position compared to all other possible positions. There is no limit to the number of variable modifications defined for the PEAKS search.

For the evaluation of search engine performance, an FPOP hemoglobin–haptoglobin data set from an experiment previously performed in our lab^32,33^ was selected for the study. The data set is available on ProteomeXchange^34^ (PXD021621). It represents a complex proteomic sample with features such as disulfide bonds and glycosylations, which must be taken into account during the sample treatment.^35^ Steps such as carbamidomethylation of Cys and deglycosylation alter the peptide features and increase the complexity of the sample, raising the bar for search engines.

## Methods

### Data Analysis

Data were searched against a database consisting of sequences of Hb (Uniprot IDs P69905 and P68871) and Hp (Uniprot ID P00738) supplemented with a deglycosylated form of β subunit, together with 246 sequences of common contaminants (including trypsin and catalase), using Mascot Server 2.7.0 software (Matrix Science Inc., Boston, MA, USA) and PEAKS Studio X+ software (Bioinformatics Solutions Inc., Waterloo, ON, Canada). The MGF files for Mascot were generated in Compass DataAnalysis v.5.3 (Bruker Daltonics, Billerice, MA, USA). The database was infused with reverse sequence decoys. Only tryptic peptides with up to two misscleavages were allowed. The tolerance of the precursor ions was set at 12 ppm, and the mass tolerance for the MS/MS fragment ions was set at 0.05 Da. The significance threshold was set to *p* ≤ 0.05. The considered variable modifications are listed in Table S1. Only peptides with a single FPOP modification were considered for the analysis. This condition was applied because the FPOP modifications might alter the protein’s structure, which could make solvent accessible areas that are not present in the native state of the protein. Peptide-spectrum matches were cut off by default values: −10log_10_ (*P*) ≥ 2 for Mascot and −10log_10_ (*P*) ≥ 15 for PEAKS. The intensities of the peptides were determined using Compass DataAnalysis v.5.3.

Due to the fact that Mascot’s results were obtained as a list of all identifications, the modified peptides with multiple IDs were clustered in a 30 s retention time window, from which the best scoring ID was selected.

The intersections of the modifications detected within the replicates and between the Mascot and PEAKS results were visualized using Venn diagrams using the “matplotlib_venn”^36^ Python package.

The results were subjected to Principal Component Analysis (PCA), where the parameters evaluated were score, site probability (MD-score for Mascot and AScore for PEAKS), and precursor intensity. For the purpose of linearization, log_10_ (AScore) was used for the PEAKS results. The PCA and subsequent visualization of the results have been carried out in R^37^ using the “FactoMineR”,^38^ “factoextra”,^39^ and “corrplot”^40^ packages.

## Results and Discussion

### Modifications Detected within Replicates

Using Mascot and PEAKS, a peptide search was done separately for each replicate. The resulting lists of IDs only partially overlapped (Figure S1). In the hemoglobin (Hb) sample, Mascot identified a total of 603 modified peptides with 154 IDs being mutual for all three replicates (Table 1). In the haptoglobin (Hp) sample, a total of 407 modified peptides were identified, of which 107 were mutual. In the hemoglobin–haptoglobin (HbHp) complex sample, 1148 modified peptides were identified, of which 309 were mutual. As for PEAKS, the search of the Hb sample yielded a total of 588 modified peptides, of which 119 were mutual for all three replicates. For the Hp sample, 525 modified peptides were identified, of which 114 were mutual. In the sample of the HbHp complex, a total of 1216 modified peptides were identified, with 277 of them being mutual.

**Table 1:** Modified Peptides in Hb, Hp, and HbHp Complex Samples Identified by Mascot and PEAKS

	Mascot			PEAKS		
	all	mutual		all	mutual	
Hb	603	154	25.5%	588	119	20.2%
Hp	407	107	26.3%	525	114	21.7%
HbHp	1148	309	26.9%	1216	277	22.8%

The portion of mutual IDs to the total amount of IDs was higher in the case of Mascot, around 26%, in contrast to PEAKS, which averaged around 22%. This shows a higher consistency in the identification of modified peptides by Mascot. The Mascot results include occurrences of the same modified peptide (meaning type and position) from different retention times. This proves to be interesting, especially for encompassing all possible variants of modified peptides, which could reside on different atoms of the amino acid side chain, resulting in elution of the same peptide at multiple retention times (isobaric forms of modifications).^41^

For further evaluation, only IDs detected in all three replicates were accepted.

### Intersection between Mascot and PEAKS

**Figure 1:**
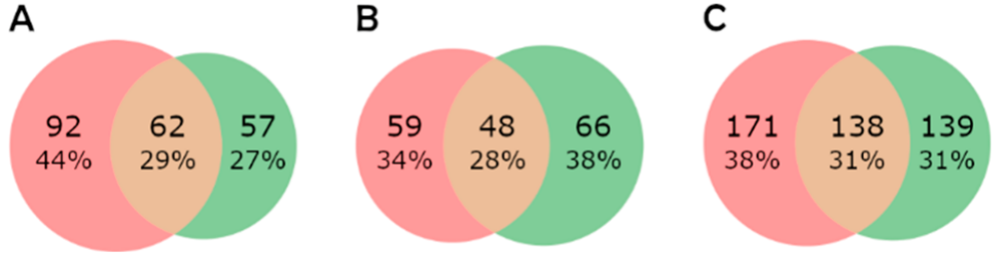
Intersections (orange) of IDs by Mascot (red) and PEAKS (green) in the (A) hemoglobin sample, (B) haptoglobin sample, and (C) HbHp complex sample.

Considering only modified peptides identified in all 3 replicates, a comparison between Mascot and PEAKS was made. Matching IDs possessed the same type, site of modification, and retention time. Only a limited number of identified modified peptides were shared among search engines (Figure 1). It is worth noting that the proportion of mutual IDs remains the same for analyses of single peptides as well as for the HbHp complex. This suggests that the IDs done by search engines are not related to the complexity of the data set but to the logic of identification.

The observed mutual exclusivity might be explained to some extent due to the Mascot search engine being limited in the number of possible defined variable modifications to 9. Based on our prior evaluation of the current data set,^32,33^ together with previously published data processing setups^20^ and described rates of reactivity,^14,15^ the His → Asn, His → Asp, and trioxidation modifications were defined for the PEAKS search only (Table S1). His already has 4 different modifications defined, and we were more interested in Met −32 Da, as a modification of the most reactive residue. Arg deguanidination was included for both search engines to widen the spatial resolution of the method. Trioxidation was not used in Mascot, because it is an extensive modification occurring mainly on Cys that requires the reaction of 3 radicals,^15^ reducing the relevance for structural information. Moreover, the inclusion of positional isomers in the Mascot results (mentioned above) further increases the number of unique IDs. The modifications, which were defined only in PEAKS searches, make up around one-third of the exclusive IDs. The setup of a PEAKS search with the same set of modifications as Mascot was tested, but the number of mutual IDs did not increase. Another reason for the differences in IDs could originate from fragmentation spectra, where the ions do not cover the exact position of the modification, and the precise position cannot be determined. In these cases, each of the search engines could tend to a different localization of the modification. Lastly, the results suggest that the search engines differ in the matter of identified modifications, with Mascot generally being able to detect more oxidations, carbonyls, and His +5 Da over PEAKS (Table 2).

**Table 2:** Types of Modifications Detected by Mascot and PEAKS in the HbHp Complex

	Mascot		PEAKS	
oxidation	144	47%	112	40%
dioxidation	36	12%	30	11%
carbonyl	61	20%	42	15%
decarboxylation	22	7%	21	8%
Met –32 Da	7	2%	4	1%
His +5 Da	19	6%	9	3%
His –10 Da	16	5%	10	4%
Arg deguanidination	4	1%	5	2%
His → Asp^a^	NA		13	5%
His → Asn^a^	NA		14	5%
trioxidation^a^	NA		17	6%

a Only defined for search in PEAKS.

### Principal Component Analysis

Principal Component Analysis (PCA) was employed to further analyze the results of the selected search engines using the PSM score, the probability of the modification site, and the intensity of the peptide as criteria. The PCA revealed the grouping of IDs into distinct clusters and relations among the selected criteria. For each condition (Hb, Hp, and HbHp), the PCA yielded 3 dimensions, with the representation of supplied variables (Score, Site, and Intensity) being similar across conditions.

The PCA statistical models for hemoglobin-only and haptoglobin-only samples (Figures S2–S7) show a similar behavior (in terms of the ID’s distribution and the representation of variables) as for the HbHp complex sample. For this reason, the HbHp complex sample was selected for further examination.

In the case of the HbHp complex, a statistical evaluation was performed for modified peptides of both proteins listed together. The results obtained by Mascot contained 175 modified peptides of Hb and 134 modified peptides of Hp. The first two dimensions yielded by PCA explained 44.4% and 32% of the data set variability, respectively. Dimension 1 represents mainly the Score and Intensity (both by roughly 60%), and the Site is represented by 15% (Figure S8A). The Score and Intensity exhibit a 75% positive correlation with Dimension 1 (Figure S9A). This alignment indicates a very high cross-correlation between Score and Intensity. Dimension 2 represents almost 85% of Site variability, with more than 92% positive correlation of Site with Dimension 2.

**Figure 2:**
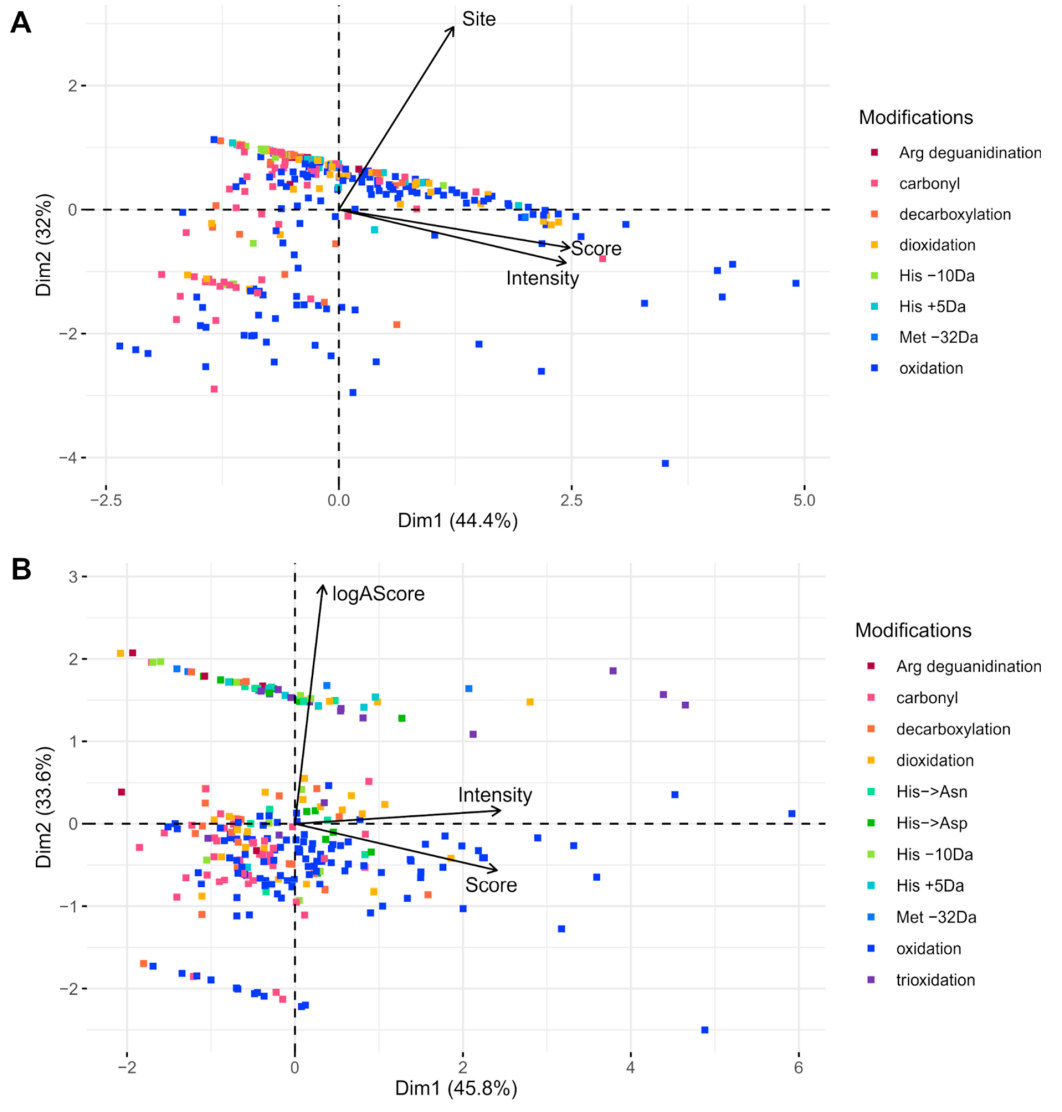
Principal component analysis of modified peptides identified in the HbHp complex sample by (A) Mascot and (B) PEAKS. The plot of color-coded modifications shows their distribution within dimensions defined by the PCA. The vectors show the correlation of the variables with the PCA dimensions. The logAScore variable represents the probability of site determination. In both cases, the IDs are distributed into 3 clusters. For Mascot, most of the IDs are in the top cluster, while for PEAKS, the majority of the IDs are in the middle cluster.

**Figure 3:**
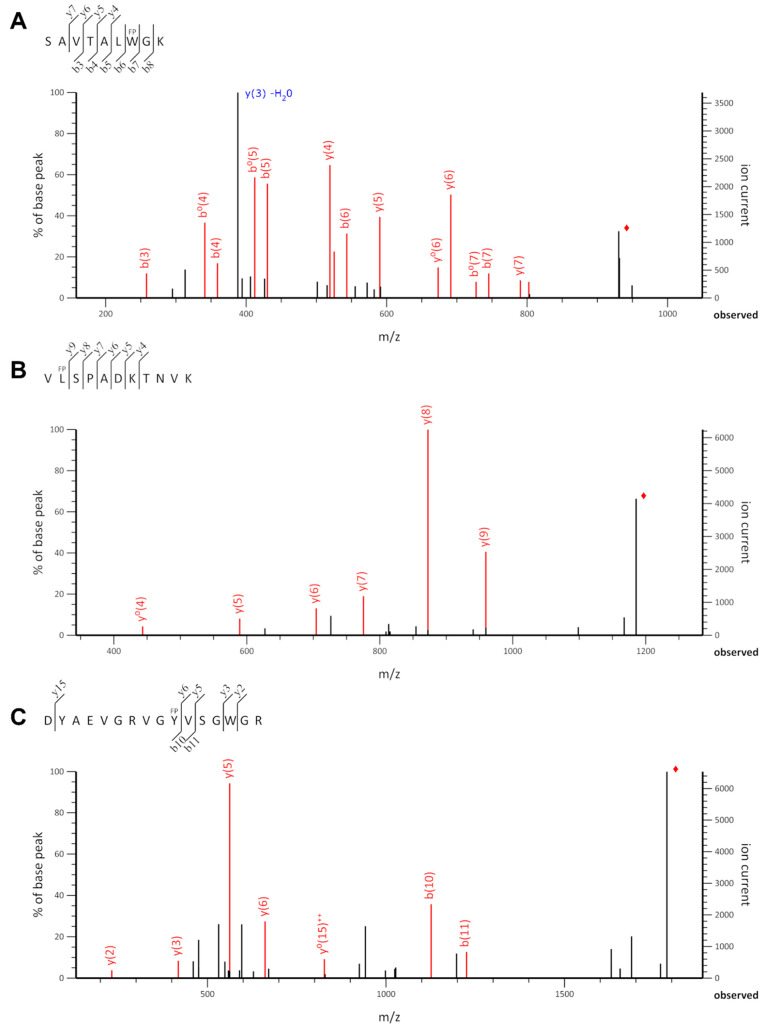
Fragmentation spectra of selected IDs from the PCA model of the Mascot search results for the HbHp complex. The ⧫ sign labels the precursor ion. (A) Trp7 oxidation (+15.995) in the peptide SAVTALWGK in its singly charged form at *m*/*z* 948.51 and RT 9.2 min from the top cluster, y3 −H_2_O undetected by Mascot was annotated manually; (B) Leu2 carbonyl (+13.979) in the peptide VLSPADKTNVK in its doubly charged form at *m*/*z* 593.33 and RT 12.5 min from the middle cluster; (C) Tyr10 oxidation (+15.995) in the peptide DYAEVGRVGYVSGWGR in its triply charged form at *m*/*z* 596.29 and RT 9.8 min from the bottom cluster. The representative IDs show that the PCA is able to separate reliable, questionable, and unreliable modified peptides.

The distribution of the modified peptides in the PCA plot (Figure 2A) shows that most of the IDs are accumulated in a horizontal cluster at the top of the plot. A closer look shows that oxidations predominate toward the right side (alongside the Score and Site vector), in contrast to the left side, which is populated by a more diverse spectrum of modifications, including carbonyls, His −10 Da, and His +5 Da. On the basis of manual validation, the modified peptides from this cluster are identified with a high certainty, which corresponds to precise coverage of the modified residue in the MS/MS spectrum. An example is Trp oxidation in a peptide with the sequence SAVTALWGK in its singly charged form at *m*/*z* 948.51 and RT 9.2 min (Figure 3A). The spectrum shows 10 fragment ions out of 16, including a b3–b8 ion series with b6 and b7 ions covering exactly the site of modification. The second linear cluster of IDs spanning the middle of the distribution shows once again that oxidations are mainly on the right side of the cluster, whereas carbonyls and dioxidations are located on the left side. The exact site of modifications falling into this group could not always be precisely determined due to an incomplete or unconvincing fragmentation spectrum. Leu carbonyl in the peptide VLSPADKTNVK in its doubly charged form at *m*/*z* 593.33 at RT 12.5 min (Figure 3B) is such a case. The spectrum contains only y-ions, with y10 missing, leaving both Val1 and Leu2 as possible sites of modification. The IDs scattered in the bottom area of the PCA plot (mainly oxidations) are subject to great uncertainty with insufficient peptide fragment coverage, for example, Tyr10 oxidation in a peptide with the sequence DYAEVGRVGYVSGWGR in its triply charged form at *m*/*z* 596.29 and RT 9.8 min (Figure 3C).

Because there is no fragmentation coverage between the y6 and y15 ions, there are 5 possible sites of modification, making this ID unusable. Given these points, the PCA model for Mascot shows a good separation of reliable, questionable, and completely unreliable IDs.

**Figure 4:**
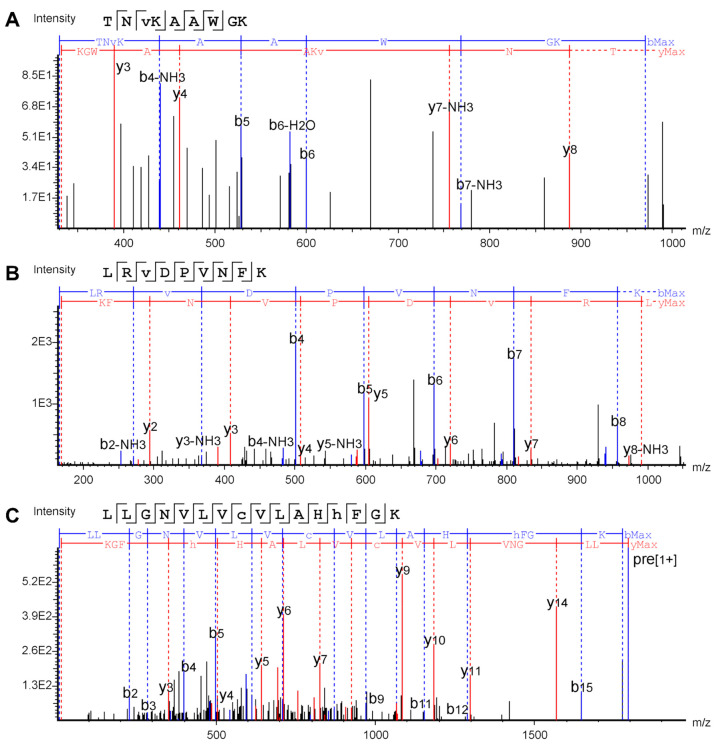
Fragmentation spectra of selected IDs from the PCA model of the PEAKS search results for the HbHp complex: (A) Val3 carbonyl (+13.979) in the peptide TNVKAAWGK in its singly charged form at *m*/*z* 988.52 at RT 7.27 min from the top cluster; (B) Val3 oxidation (+15.995) in the peptide with the sequence LRVDPVNFK in its doubly charged form at *m*/*z* 552.31 at RT 8.19 min from the middle cluster; (C) His13 oxidation (+15.995) in the peptide LLGNVLVCVLAHHFGK in its doubly charged form at *m*/*z* 897.00 at RT 21.01 min from the bottom cluster. The selected IDs show that the fragmentation coverage of the site of modification and signal intensity do not have a clear relation to the AScore and position within the PCA clusters.

As for the PEAKS search of the HbHp complex, the input list contained 150 Hb modified peptides and 124 Hp modified peptides. Dimension 1 accounts for 45.4% of the variability, and Dimension 2 accounts for 33.3%. Dimension 1 represents Score and Intensity by 68% (Figure S8B), with both variables being positively correlated with Dimension 1 by over 82%, but the Score and Intensity variables show only partial cross-correlation. One possible explanation is the additive merging of MS/MS spectra. While fragments from several scans could contribute to the PSM score, the intensity of the compound in the chromatogram might be low. Dimension 2 represented the entirety of the logAScore variability (99.97%) and was fully positively correlated (99.99%). The PCA model (Figure 2B) shows separation into 3 clusters. Manual validation has shown that the top horizontal cluster consists predominantly of IDs that are unambiguous by definition. This means that there is only one residue in the peptide sequence that could carry out the modification. For illustration, the fragmentation spectrum of Val carbonyl in the TNVKAAWGK peptide in its singly charged form at *m*/*z* 988.52 at RT 7.27 min (Figure 4A) consists of very low intensity signals. Nevertheless, this ID has been assigned the highest certainty possible. The second cluster spans around the center of origin and includes most of the IDs. This cluster predominantly consists of oxidations, carbonyls, decarboxylations, and dioxidations. Closer inspection revealed that most of these IDs have a low AScore, although the fragment coverage seems sufficient.

For example, the fragmentation spectrum of Val3 oxidation in a peptide with the sequence LRVDPVNFK in its doubly charged form at *m*/*z* 552.31 at RT 8.19 min (Figure 4B) contains 14 out of 16 fragment ions; yet, the assigned AScore is 20.41. The third, linear cluster at the bottom consists of IDs with absolute uncertainty of position (with AScore = 0), although the fragmentation spectra might cover the entire peptide sequence. For instance, His13 oxidation in the LLGNVLVCVLAHHFGK peptide in its doubly charged form at *m*/*z* 897.00 at RT 21.01 min (Figure 4C), whose fragmentation spectrum contains almost all possible fragments. Our explanation is that the merged spectrum contains a number of low-intensity signals, and the fragment ions determining the oxidation position to be His13 are among those low-intensity signals (y3, y4, b11, b12). Additionally, in the sequence scheme on the top, PEAKS indicates that there is supposedly a b14 fragment, but such a signal is not present in the spectrum. Furthermore, after manual validation of the spectrum, we were able to annotate ions 367.2, which corresponds to y3 carrying oxidation, and 1081.44, which corresponds to y9 carrying carbonyl. Thus, sometimes the AScore cannot be confident. These results show that, even though the PEAKS IDs are separated by PCA into distinct clusters, the relation to certainty in site determination is inconclusive. From the modified peptides identified by both Mascot and PEAKS, 4 representatives have been selected for comparison. First, Met15 oxidation in a peptide with the sequence VADALTNAVAHVDDMPNALSALSDLHAHK in its triply charged form at *m*/*z* 1004.84 and RT 16.61 min is a highly probable and extensive modification. Both search engines were able to identify numerous fragmentation ions (Figure S10) and thus assign a very high score (Mascot: 153.34; PEAKS: 105.68), but PEAKS did not account for relatively strong y14 and b18 ion signals assigned by Mascot. Furthermore, Mascot evaluated the certainty of oxidation localization as almost 100%, determining it to be located in the top cluster of the PCA plot. PEAKS, on the other hand, evaluated its certainty only as AScore 11.12, which ranks among the lower ones and means localization to the lower part of the middle cluster.

Second, the His11 carbonyl from the peptide with the sequence VVAGVANALAHK in its doubly charged form at *m*/*z* 582.33 and RT 9.44 min represents a low abundance modification. The identification done by Mascot comes from ions b4, y5, and y7–y10, which do not cover the site of modification (Figure S11A). The assigned score of 32.1 is among the lower ones. The coverage is not sufficient to differentiate between positions Leu9 and His11, yet Mascot sides with the latter, signaling 49.82% certainty, ranking within the middle cluster of the PCA plot. His11 carbonyl identified by PEAKS relies on the fragmentation spectrum with numerous clear y-ion signals, together with several b-ions on the level of noise (Figure S11B), resulting in a middle tier score of 71.05. Although PEAKS shows almost AA-level fragmentation sequencing, AScore 14.04 indicates not very high certainty of the position and localization to the lower part of the middle cluster.

Val5 oxidation in the VVAGVANALAHK peptide in its doubly charged form at *m*/*z* 583.34 at RT 5.9 min is the third example. In this case, Mascot assigned a high score of 102.79, while PEAKS assessed the identification at a score of 60.44, which represents the low end of the evaluation scale for PEAKS. The fragmentation spectrum used for identification by Mascot contains y-ions only, with just y1 and y8 ions missing (Figure S12A). Since the y7 and y9 ions border the Gly-Val residues and the oxidation is not defined for Gly, Mascot gives a site determination confidence of 99.99%, ranking as the top cluster ID. The identification by PEAKS relies on 10 (out of 11) y-ions and 8 b-ions (Figure S12B). Although the PSM score assigned by PEAKS is among the lower ones, the fragmentation coverage is sufficient to give the AScore of 26.57, meaning middle cluster in the PCA.

Finally, the Glu5 decarboxylation in the peptide KQLVEIEK in its monocharged form at *m*/*z* 956.58 at RT 5.43 min represents identification, which has a low score from both search engines (Mascot: 23.72; PEAKS: 50.06). The fragmentation spectrum for identification done by Mascot (Figure S13A) lacks precise coverage of the site of modification, with only low intensity b6-H_2_O and b6-NH_3_ ions differentiating between the Glu5 and Glu7 positions. This results in 68.98% confidence in site determination and localization to the lower part of the top cluster. The fragmentation spectrum for PEAKS identification (Figure S13B) contains 11 out of 14 possible fragments, but the signals for b2, b5, b6, y4, and y5 have a borderline noise-level intensity. The site determination is evaluated with a fairly confident AScore 24.32, meaning middle cluster.

These results demonstrate that Mascot is able to assess confident site determination for modifications, even in cases of a lower PSM score. On the other hand, a high PSM score of PEAKS IDs does not guarantee a reliable determination of the modification site. Similarly, IDs with lower PSM score can still result in a reliable localization of the modification. From a user point of view, Mascot assignments have a clear relation to the fragmentation coverage of the modification position, while the AScore value does not provide reliable guidance on the certainty of modification localization. The AScore evaluation appears to be low, even in cases with a high fragmentation coverage. Additionally, the highest possible confidence (AScore of 1000) is reachable only for unambiguously defined modifications. Furthermore, the additional merging performed by PEAKS yields its identifications and classification inconclusive, as there is little to no option to confirm PEAKS identifications against the raw data.

## Conclusions

The results of this study show that the selection of the search engine for FPOP data evaluation has a significant impact on the detected modifications. We compared the consistency of selected search engines within replicates and compared results of the search engines with each other, where Mascot shows greater consistency over PEAKS. The number of identifications detected by both search engines was limited (31%). Principal Component Analysis revealed distinct clusters of modified peptides based on their scores, site probabilities, and intensities. Mascot identifications show a more confident site determination and a clear relationship between the quality of the fragmentation spectrum and the assigned site confidence. From the point of view of data evaluation, Mascot requires less manual validation. Most of the IDs belong to the top cluster and are reliable. Manual validation of the IDs from the middle cluster is advisable, with oxidations generally scoring better than carbonyls. Modifications below the middle cluster should be discarded. Clustering of PEAKS IDs does not relate clearly to the reliability of identification, which leads to the need for manual validation of the majority of the results. Furthermore, data refinement performed by PEAKS prevents backchecking with raw data.

Overall, this study provides insights into the performance and differences between Mascot and PEAKS in the analysis of FPOP data. We conclude that using Mascot for FPOP data leads to more reliable and understandable IDs and requires less manual validation. Still, there is the need for further development and automatization of the data processing workflow for FPOP analysis.

## Supporting Information Available

Defined variable modifications (Table S1), intersections of IDs for Hb and Hp samples (Figure S1), quality of variable representation for PCA of Hb sample (Figure S2), variable correlations for PCA of Hb sample (Figure S3), PCA for Hb sample (Figure S4), quality of variable representation for PCA of Hp sample (Figure S5), variable correlations for PCA of Hp sample (Figure S6), PCA for Hp sample (Figure S7), quality of variable representation for PCA of HbHp sample (Figure S8), variable correlations for PCA of HbHp (Figure S9), and fragmentation spectra of example modified peptides (Figures S10–S13) (PDF)

## Abbreviations

FPOP: Fast Photochemical Oxidation of Proteins
Hb: hemoglobin
Hp: haptoglobin
HbHp: hemoglobin–haptoglobin
PSM: peptide-spectrum match
RT: retention time (min)
