# Supplementary material for: Quantifying the Impact of the Peptide Identification Framework on the Results of Fast Photochemical Oxidation of Protein Analysis
Source: J Proteome Res. 2023 Dec 29;23(2):609–17. doi: 10.1021/acs.jproteome.3c00390 (PMC10845142; doi:10.1021/acs.jproteome.3c00390)
Supplement: Supplementary file 1 — pr3c00390_si_001.pdf [file pr3c00390_si_001.pdf]

Supplementary data

Quantifying the Impact of the Peptide Identification Framework on the Results of Fast Photochemical Oxidation of Proteins Analysis

Marek Zakopcanik[1,2], Daniel Kavan[1], Petr Novak[1], Dmitry S. Loginov[*,1]

[1]Institute of Microbiology, The Czech Academy of Sciences, 14220 Prague, Czech Republic

[2]Faculty of Science, Charles University, 12843 Prague, Czech Republic

*Email: dmitry.loginov@biomed.cas.cz

List of Supplementary data

Defined variable modifications (Table S1), intersections of IDs for Hb and Hp samples (Figure S1), quality of variables representation for PCA of Hb sample (Figure S2), variables correlation for PCA of Hb sample (Figure S3), PCA for Hb sample (Figure S4), quality of variables representation for PCA of Hp sample (Figure S5), variables correlation for PCA of Hp sample (Figure S6), PCA for Hp sample (Figure S7), quality of variables representation for PCA of HbHp sample (Figure S8), variables correlation for PCA of HbHp (Figure S9), fragmentation spectra of example modified peptides (Figures S10 – S13).

Table S1. Overview of defined variable modifications.

**modification name**	**mass difference (Da)**	**amino acid**
carbamidomethylation	+57.02146	C
FPOP oxidation	+15.994915	F, H, I, K, L, M, P, R, V, W, Y
FPOP dioxidation	+31.989828	C, F, M, W, Y
FPOP carbonyl	+13.979265	E, H, I, L, P, Q, R, V
FPOP decarboxylation	-30.010565	E, D
FPOP Met-32Da	-32.008457	M
FPOP His+5Da	+4.9789	H
FPOP His-10Da	-10.031968	H
FPOP Arg deguanidination	-43.053432	R
FPOP trioxidation*	+47.984745	C, W
FPOP His->Asp*	-22.03197	H
FPOP His->Asn*	-23.01598	H

* only defined for search in PEAKS

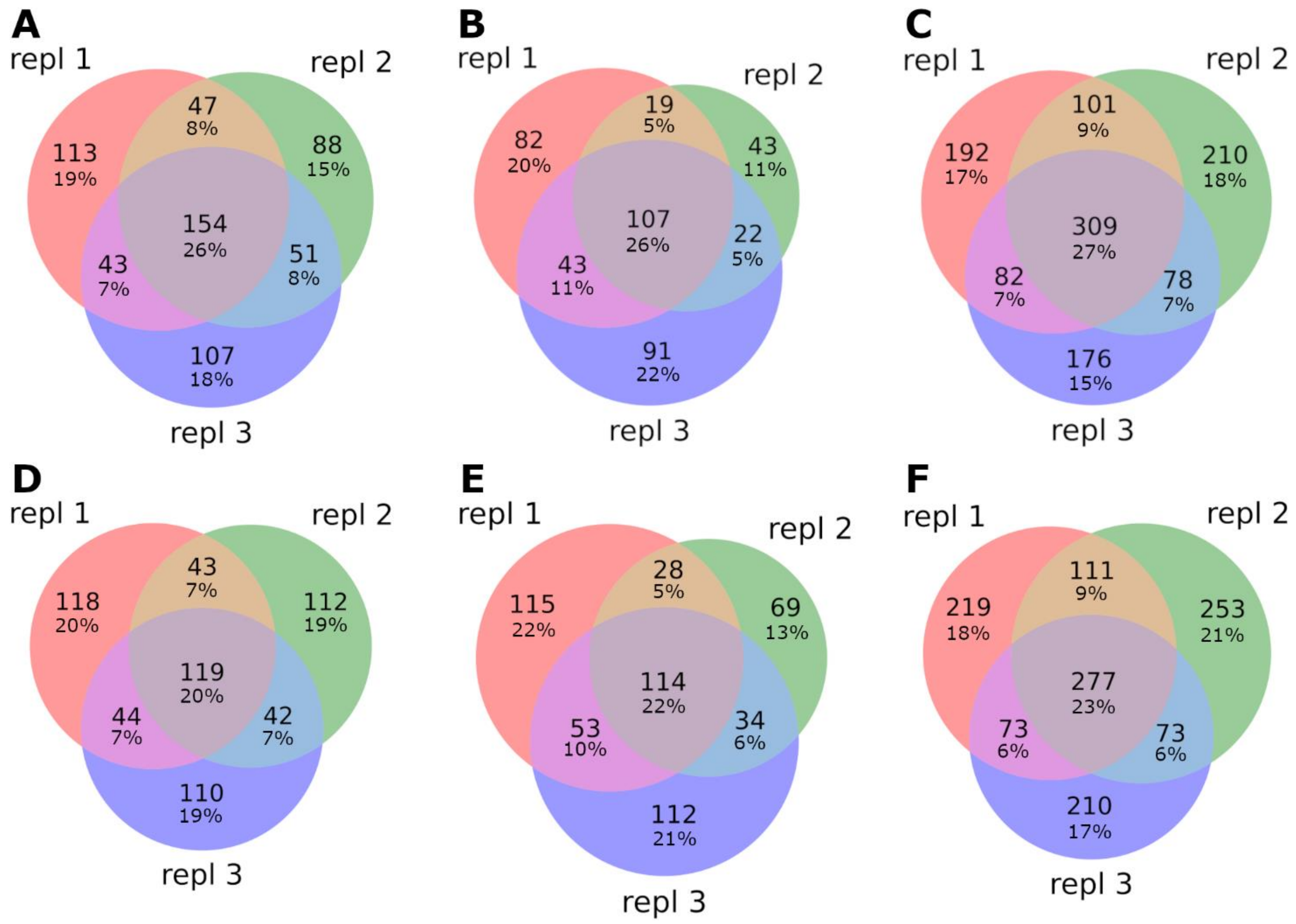

Figure S1. Intersections of IDs between replicates for Mascot searches of (A) Hb; (B) Hp; (C) HbHp complex samples; and for PEAKS searches of (D) Hb; (E) Hp; (F) HbHp complex samples. Mascot exhibits higher consistency within replicates.

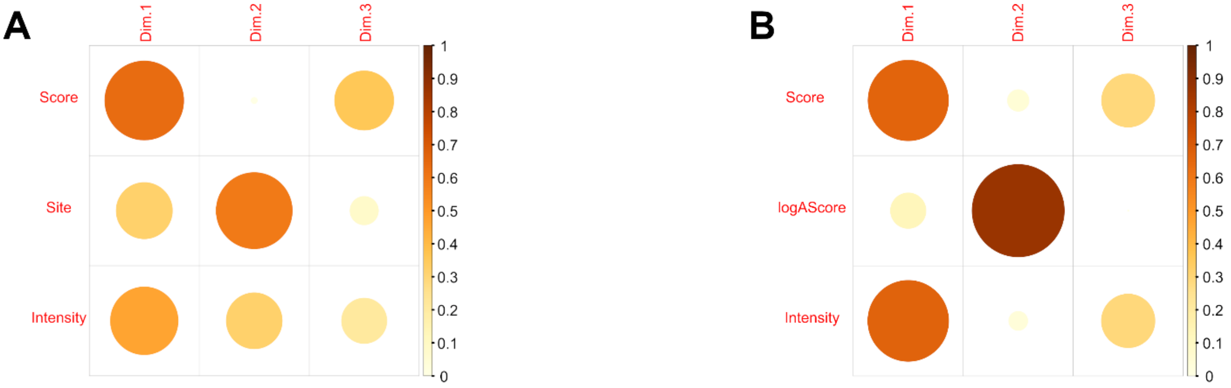

Figure S2. Quality of representation of the selected variables by the dimensions of the PCA for Hb sample searched by (A) Mascot, and (B) PEAKS. The extent of representation is visualized by both size and colour.

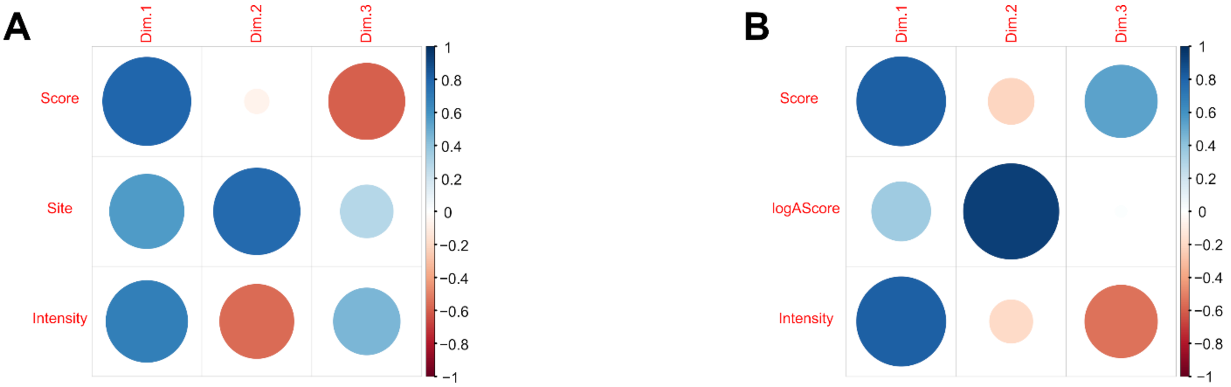

Figure S3. Correlation of the selected variables and the dimensions of the PCA for Hb sample searched by (A) Mascot, and (B) PEAKS. The extent of correlation is visualized by size and the trend of correlation as colour.

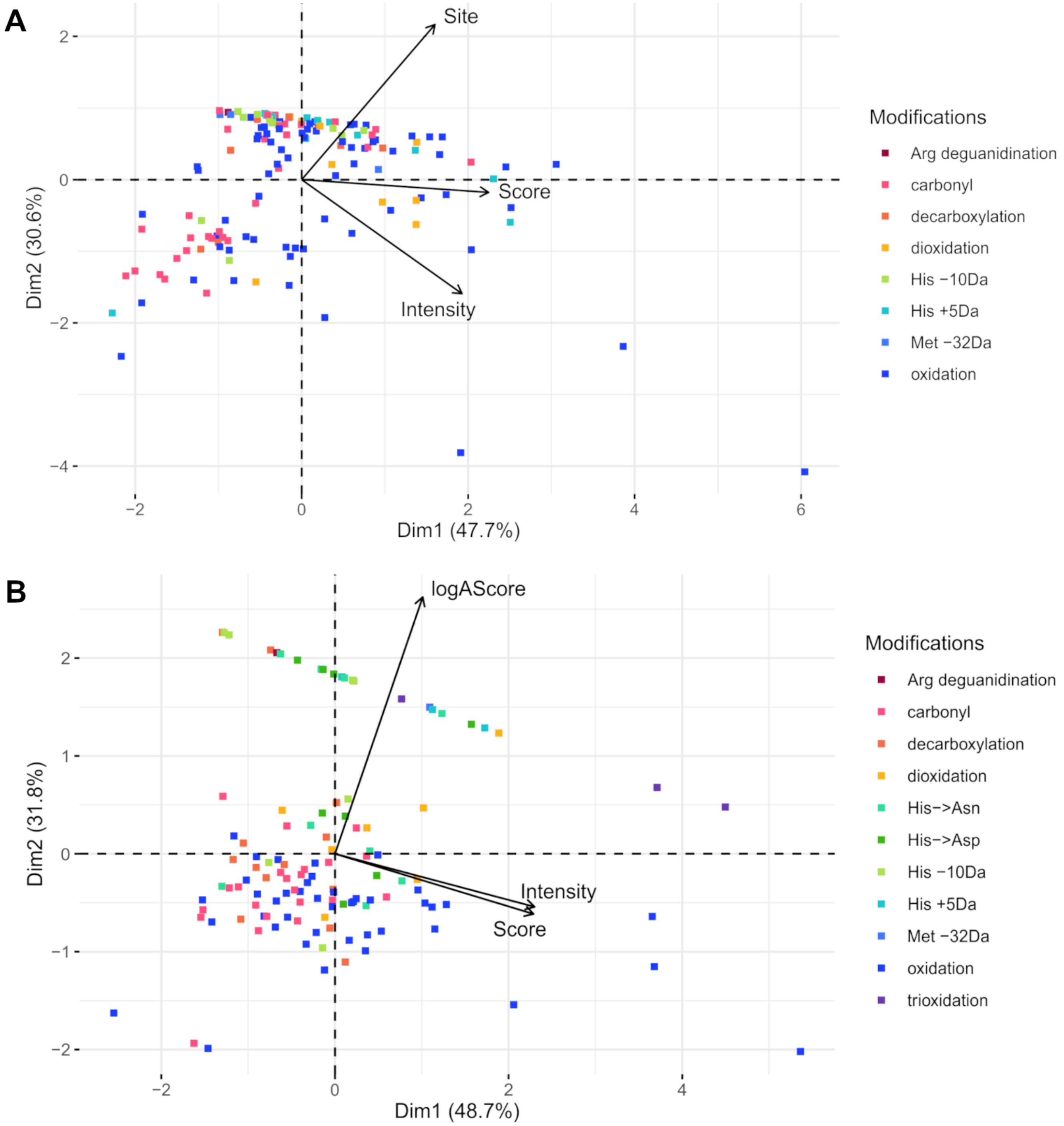

Figure S4. Principal component analysis of modifications identified in Hb sample by (A) Mascot and (B) PEAKS. Plot of color-coded modifications shows their distribution within dimensions defined by PCA. The vectors show correlation of variables with the PCA dimensions. The logAScore variable represents probability of site determination.

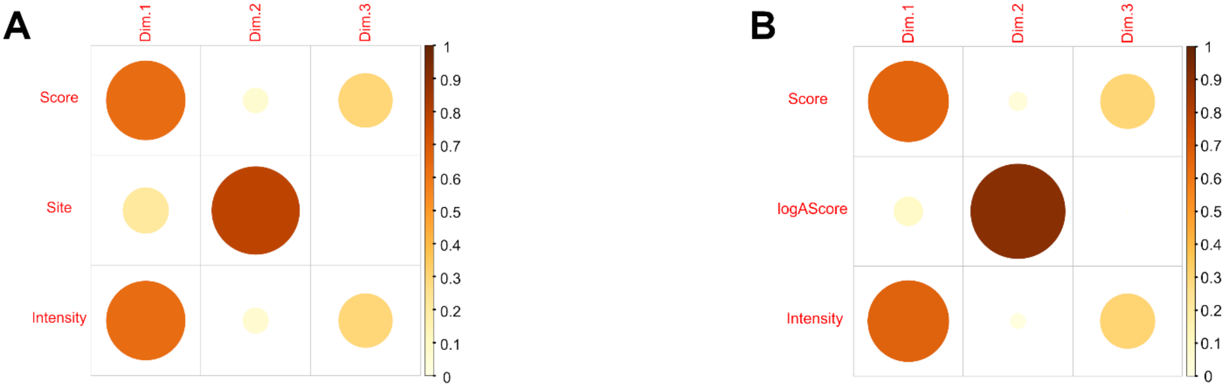

Figure S5. Quality of representation of the selected variables by the dimensions of the PCA for Hp sample searched by (A) Mascot, and (B) PEAKS. The extent of representation is visualized by both size and colour.

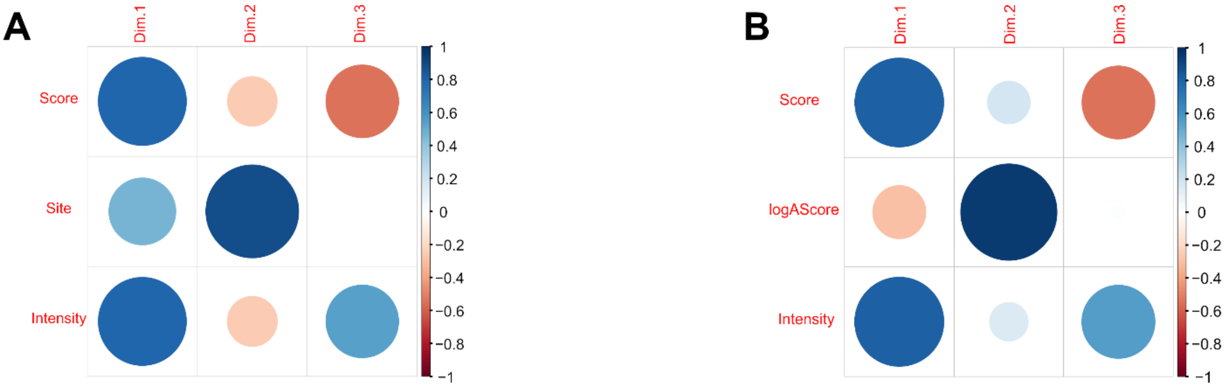

Figure S6. Correlation of the selected variables and the dimensions of the PCA for Hp sample searched by (A) Mascot, and (B) PEAKS. The extent of correlation is visualized by size and the trend of correlation as colour.

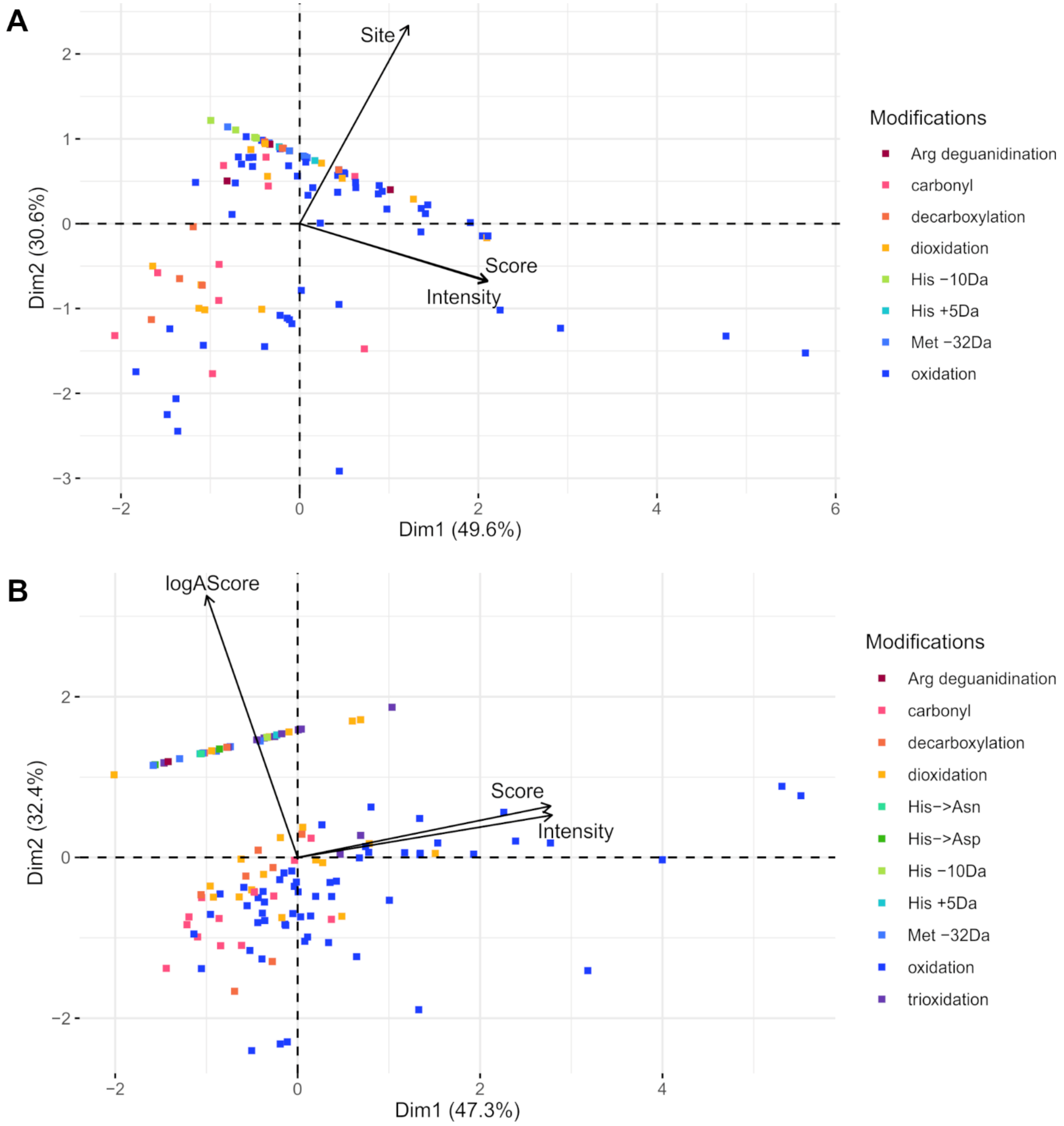

Figure S7. Principal component analysis of modifications identified in Hp sample by (A) Mascot and (B) PEAKS. Plot of color-coded modifications shows their distribution within dimensions defined by PCA. The vectors show correlation of variables with the PCA dimensions. In (A) the Score and Intensity vectors overlap. The logAScore variable represents probability of site determination.

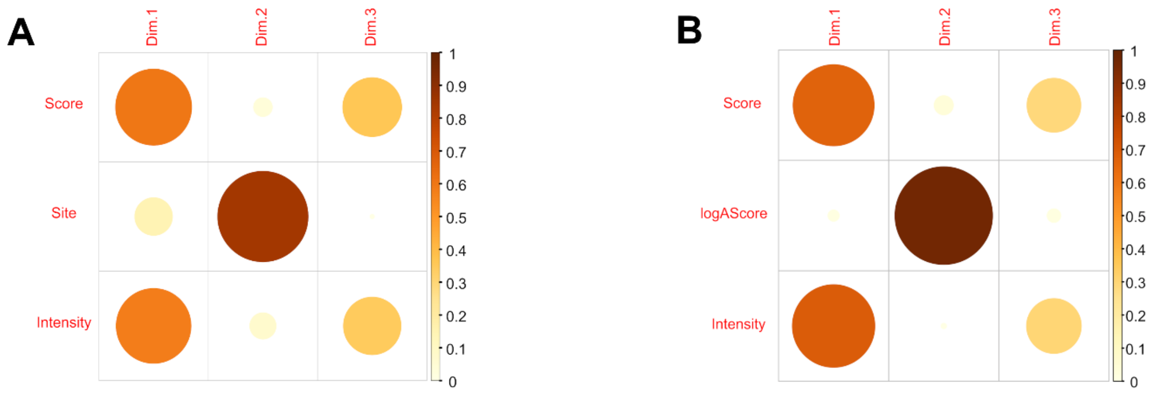

Figure S8. Quality of representation of the selected variables by the dimensions of the PCA for (A) Mascot search and (B) PEAKS search of the HbHp complex sample. The extent of representation is visualized by both size and colour.

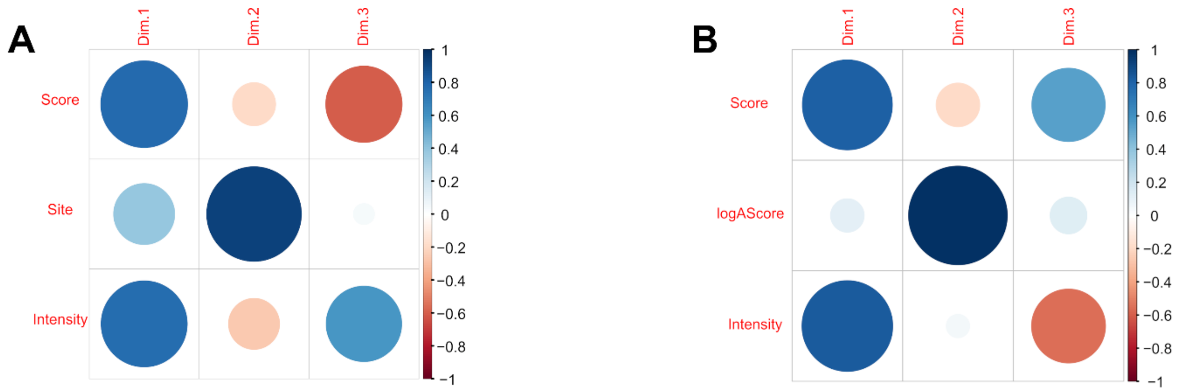

Figure S9. Correlation of the selected variables and the dimensions of the PCA for HbHp complex sample for (A) Mascot and (B) PEAKS. The extent of correlation is visualized by size and the trend of correlation as colour.

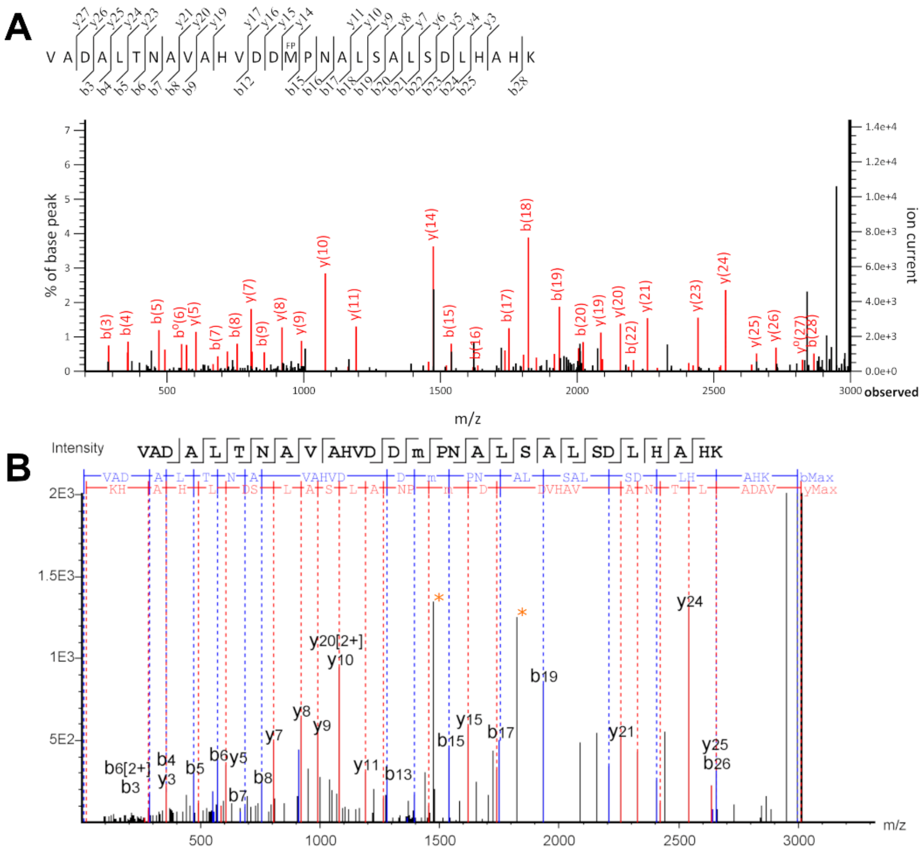

Figure S10. Fragmentation spectra of Met15 oxidation (+15.994915) in the peptide VADALTNAVAHVDDMPNALSALSDLHAHK in its triply charged form at m/z 1004.84 and RT 16.61 from the HbHp complex sample as identified by (A) Mascot (Site: 98.26%), and (B) PEAKS (AScore: 11.12). PEAKS did not account for relatively strong y14 and b18 ions (labelled *). Although the fragmentation coverage is almost complete for both search engines, Mascot shows high confidence, while PEAKS gives rather low AScore.

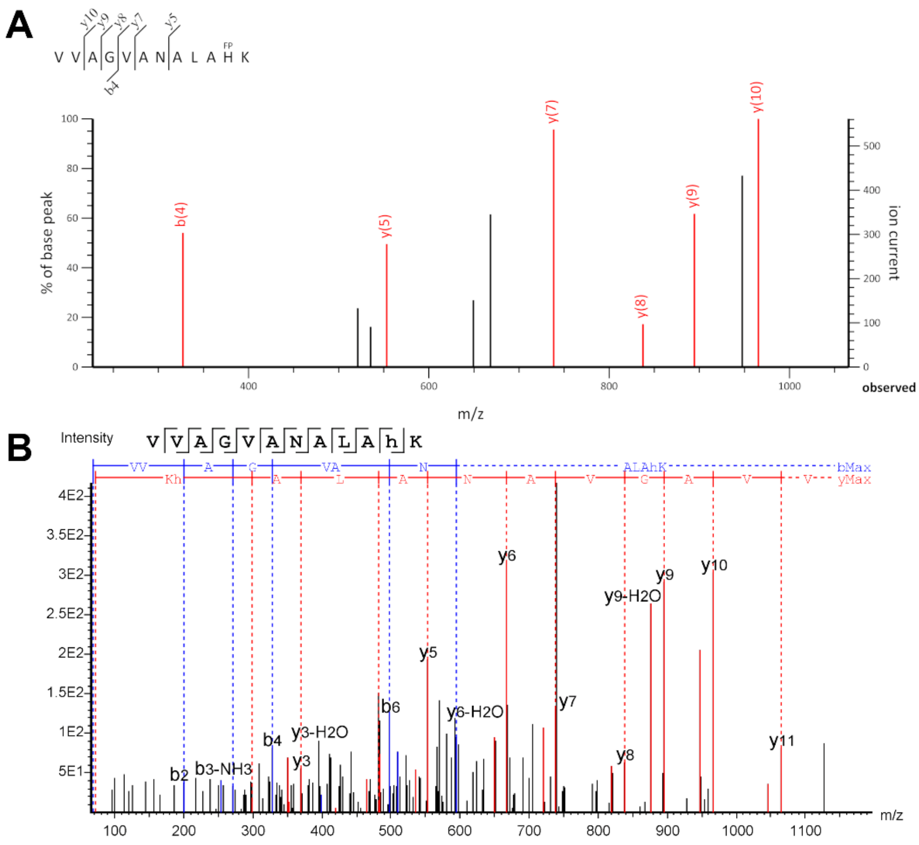

Figure S11. Fragmentation spectra of His11 carbonyl (+13.979265) from peptide VVAGVANALAHK in its doubly charged form at m/z 582.33 and RT 9.44 from the HbHp complex sample as identified by (A) Mascot (Site: 49.82%), and (B) PEAKS (AScore: 14.04). PEAKS shows better fragmentation coverage than Mascot, yet the certainty of site determination is low.

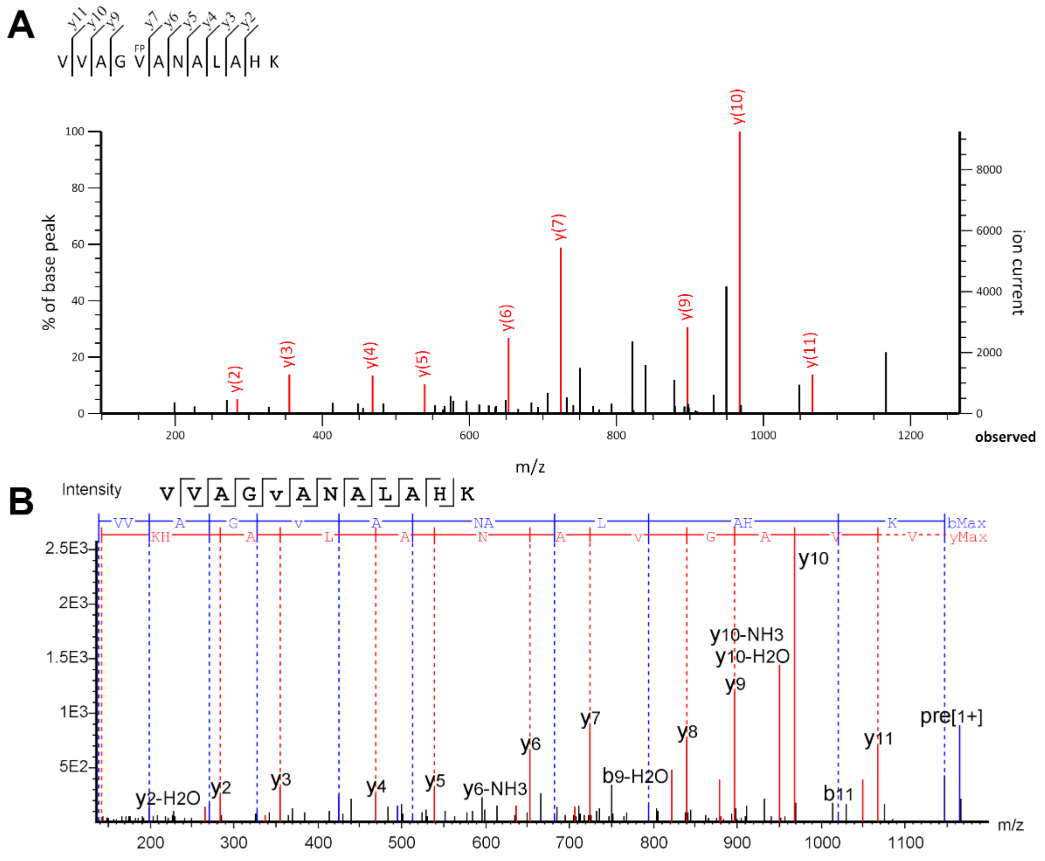

Figure S12. Fragmentation spectra of Val5 oxidation (+15.994915) in the peptide VVAGVANALAHK in its doubly charged form at m/z 583.34 at RT 5.9 from the HbHp complex sample as identified by (A) Mascot (Site: 99.99%), and (B) PEAKS (AScore: 26.57). For high intensity fragmentation spectra, both Mascot and PEAKS are able to determine the site of modification with high confidence.

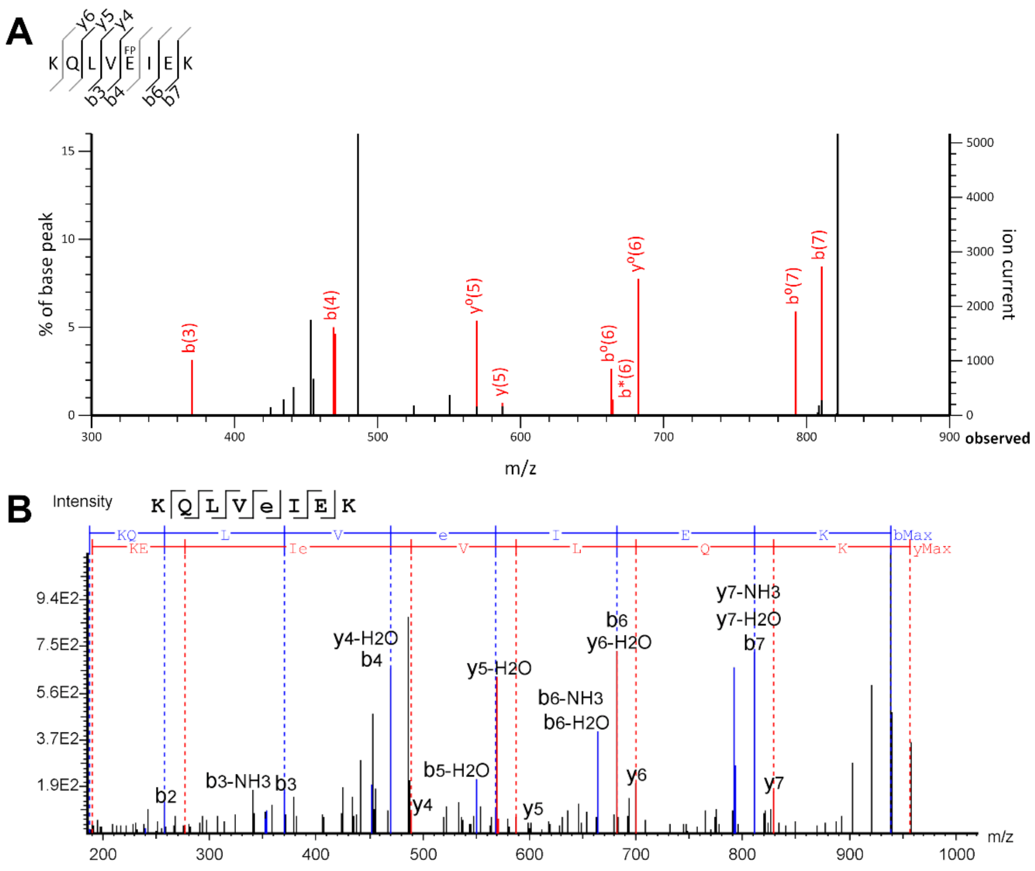

Figure S13. Fragmentation spectra of the Glu5 decarboxylation (-30.010565) in peptide with the sequence KQLVEIEK in its mono charged form at m/z 956.58 at RT 5.43 from the HbHp complex sample as identified by (A) Mascot (Site: 68.98%), and (B) PEAKS (AScore: 24.32). Even though only low intensity ions differentiate between the Glu5 and Glu7 positions, both search engines are assigning confident site determination.
